# Effect of Catechol-O-Methyltransferase Genotype Polymorphism on Neurological and Psychiatric Disorders: Progressing Towards Personalized Medicine

**DOI:** 10.7759/cureus.18311

**Published:** 2021-09-27

**Authors:** Kosha Srivastava, Olive Ochuba, Jasmine K Sandhu, Tasnim Alkayyali, Sheila W Ruo, Ahsan Waqar, Ashish Jain, Christine Joseph, Sujan Poudel

**Affiliations:** 1 Neurology, California Institute of Behavioral Neurosciences & Psychology, Fairfield, USA; 2 Internal Medicine, California Institute of Behavioral Neurosciences & Psychology, Fairfield, USA; 3 Obstetrics and Gynecology, California Institute of Behavioral Neurosciences & Psychology, Fairfield, USA; 4 Pathology, California Institute of Behavioral Neurosciences & Psychology, Fairfield, USA; 5 General Surgery Research, California Institute of Behavioral Neurosciences & Psychology, Fairfield, USA; 6 Family Medicine, California Institute of Behavioral Neurosciences & Psychology, Fairfield, USA; 7 Urology and Obstetrics & Gynecology, California Institute of Behavioral Neurosciences & Psychology, Fairfield, USA; 8 Psychiatry and Behavioral Sciences, California Institute of Behavioral Neurosciences & Psychology, Fairfield, USA; 9 Division of Research & Academic Affairs, Larkin Community Hospital, South Miami, USA

**Keywords:** psychiatric disorders, neurological disorders, comt genotype polymorphism, comt gene, catechol-o-methyltransferase

## Abstract

Different polymorphisms of the catechol-O-methyltransferase (COMT) gene affect the COMT enzyme activity. The COMT enzyme plays a major role in the pathophysiology of various neurological and psychiatric disorders. This review article aims to discuss what recent research has discovered about the association of COMT genotype polymorphism with neurological and psychiatric disorders and the scope for the knowledge to be applied for advancement in therapeutics. We searched PubMed and Google Scholar databases and found 1656 articles. We included observational studies, clinical trials, and meta-analyses in the English language published between 2019 and 2021. We screened the articles based on the title and the abstract and found 26 relevant articles. Diseases or conditions studied primarily were schizophrenia, Parkinson’s disease, Alzheimer’s disease, substance use, and depression. This article highlights how genetics influences the susceptibility of an individual to neurological and psychiatric diseases and the variations in the specific symptoms of those diseases. The review showed that the variability in individual response to therapeutic interventions stems from the gene level. This knowledge can contribute towards the dawn of a new era of personalized medicine.

## Introduction and background

Globally, there is a great burden of neurological and mental disorders. In 2005, neurological disorders contributed to 92 million disability-adjusted life years (DALYs), and this number is projected to rise by around 12% to 103 million in 2030, according to a report by the World Health Organization (WHO). Alzheimer’s disease and other dementias are expected to increase by 66% from the year 2005 to 2030. In 2005, out of the total global burden of disease, neurological disorders contributed to 10.9% in high-income countries, 6.7% in upper-middle, 8.7% in lower-middle, and 4.5% in low-income countries. Years of healthy life lost as a result of disability (YLDs) associated with Alzheimer’s disease and other dementias are expected to rise by 38% in 2030 [1]. Around 264 million people in the world are suffering from depression, and around 20 million are suffering from schizophrenia [2]. Considering how dire the situation is, it is imperative for us to deeply understand the pathophysiology of these diseases to be able to come up with better treatment and management plans. The current focus of research is on various genes contributing to these disorders.

**Figure 1:**
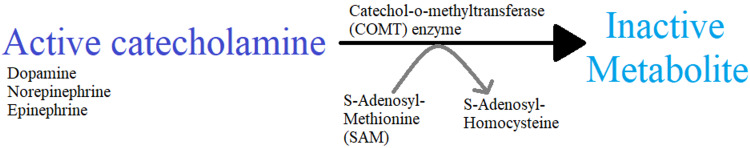
Role of catechol-O-methyltransferase in catecholamine metabolism Original diagram showing the metabolism of catecholamine by the catechol-o-methyltransferase enzyme.

Catechol-O-methyltransferase (COMT) is an enzyme that catalyzes the methylation of catechol substrates during the metabolism of catecholamines (mainly norepinephrine and epinephrine) within the body, as shown in Figure 1. 

A methyl group from S-adenosyl-L-methionine (SAM) is transferred to a catechol substrate with the help of COMT and Mg2+. O-methylated catechol and S-adenosyl-L-homocysteine (SAH) are formed as by-products of this reaction. COMT is an intracellular enzyme that has two isoforms - soluble COMT (S-COMT), which is soluble in the cytoplasm, and membrane-bound COMT (MB-COMT), which is associated with membranes [3,4]. COMT degrades more than 60% of dopamine in the prefrontal cortex and around 15% in the striatum [5]. In humans, the gene that encodes COMT is located on chromosome 22 in the band q11.21 [6]. There are seven mRNA COMT variants, formed due to insertions or deletions of primary mRNA transcript or due to splice variants, detected in the brain in low abundance [7]. Different polymorphisms of the COMT gene affect the COMT enzyme activity, which can potentially affect the pathophysiology of several neurological and psychiatric disorders. A single nucleotide polymorphism found at codon 158 that involves a transition from G to A is responsible for a change in the amino acid from Valine (Val) to Methionine (Met). COMT activity decreases four times if the Met allele is homozygous [8,9]. Three genotypes of COMT - Val/Val, Val/Met, and Met/Met display high, intermediate, and low activity of the COMT enzyme, respectively. There are several other functional COMT genotype polymorphisms present that are associated with neuropsychiatric disorders.

The association between COMT genotype polymorphism with neurological and psychiatric disorders has been actively researched in the last few decades. A recent meta-analysis comprehensively studied the association between COMT Val158Met genotype and psychiatric disorders [10]. A few neurological conditions such as migraine and Parkinson’s disease have also been studied to determine their association with COMT variants [11,12]. Recently, the focus of research has shifted towards newer concepts involving COMT gene variants and neurological and psychiatric disorders to direct research towards improved treatment strategies. Some studies have investigated the role of COMT genotype polymorphisms in response to treatment for neurological and psychiatric diseases like Parkinson’s [13] and ADHD [14]. However, more work needs to be done in this area. Additionally, better knowledge of the pathophysiology of diseases is required at the gene level for advancement in therapeutic strategies, primarily personalized or targeted strategies. In this review, we will address what current literature has discovered about the association of polymorphisms in COMT genotype with neurological and psychiatric disorders and the scope for the knowledge to be applied for advancement in therapeutics. Personalized medicine is the need of the hour in the field of neurology and psychiatry to provide better management of symptoms, improve patient care, and ultimately improve the patient’s quality of life. This will also reduce the cost of patient care in the long run.

We searched PubMed and Google Scholar databases using the keywords ‘catechol-O-methyltransferase, ‘COMT’, ‘COMT genotype’, ‘polymorphism’, ‘neurological disorders’, ‘psychiatric disorders’, and ‘mental health disorders’ and found a total of 1656 articles. We included observational studies, clinical trials, and meta-analysis studies in the English language published between 2019 and 2021. We excluded animal studies, gray literature, and all studies involving subjects below 18 years of age. After screening articles with the help of titles and abstracts, we ended up with 26 articles that were relevant to our research question. Out of these, two studies were clinical trials, 23 articles were observational studies, and one article was a meta-analysis. Out of the total number, 17 studies, including 8638 subjects, explored the association between COMT genotype polymorphism and neurological and psychiatric disorders, and nine studies were drug-based or pharmacogenetic studies that included a total of 5115 subjects. Diseases or conditions studied primarily were schizophrenia, Parkinson’s disease, Alzheimer’s disease, substance use, and depression.

## Review

Variants of COMT genotype have been extensively studied in association with various neurological and psychiatric disorders. Additionally, much focus has been seen on COMT gene variants and cognitive function. In the last few years, scientists have embarked on a journey to discover more about this association with respect to newer concepts in various neurological and psychiatric diseases and how their findings translate to advancement in therapeutic strategies moving towards personalized medicine.

Association of catechol-O-methyltransferase genotype polymorphism with psychiatric disorders

It has been long established that the dopaminergic system is involved in the pathogenesis of schizophrenia. COMT enzyme takes part in the dopamine degradation pathway and plays a vital role in schizophrenia. In the last few decades, several studies have shown an association between schizophrenia and COMT genotype polymorphisms. Recently, researchers have undertaken studies involving schizophrenia patients and COMT genotype variants, associating them with newer concepts. In 2021, a study was conducted to assess the effect of interaction between genotype polymorphisms of COMT (COMT Val158Met or rs4680) and IL-10 (IL-10 -592A/C or rs1800872) on cognition in 244 patients with chronic schizophrenia and in 396 healthy controls. The study found that IL-10 did not have any effect on cognition if measured alone; however, COMT alone had an impact on language and schizophrenia. Additionally, the study confirmed more significant interactive effects of genotypes of COMT and IL-10 on cognition in schizophrenia patients than in controls. The study revealed that the findings could be valuable for further discussion about the interactive effects of different systems on cognition in schizophrenia patients as well as for drug administration [15]. Another research was done in 2020 to study the effect of rs1076560 (dopamine receptor D2 or DRD2) and rs4680 (COMT Val158Met) on tardive dyskinesia and cognition in schizophrenia patients. The study included a total of 502 cases of schizophrenia and 448 controls. The study also investigated a subset of 80 patients positive for tardive dyskinesia and 103 negatives for the same condition. For cognition, 299 cases and 245 controls were considered. The study found that this single nucleotide polymorphism (SNP) of COMT was not associated with schizophrenia; however, rs4680 was associated with tardive dyskinesia, and it was also noted that people with the rs4680 variant who smoked had an increased risk of tardive dyskinesia. This variant also had a positive association with emotion efficiency in schizophrenia cases (P=0.04). All of these findings gave this association a pharmacological significance [16]. Another study that was done in 2019 interestingly looked at 135 chronic schizophrenia patients with a history of cannabis use and genotyped them for the Val158Met variant of COMT. The study discovered that an interaction between genotype polymorphism and cannabis use produces an effect on cognitive functions such as processing speed and verbal fluency and that the Met carriers performed better on both tasks. The seemingly converging pathways of cannabis use and COMT make it essential to assess them in developing personalized interventions and treatment strategies [17]. A neuroimaging-based study done in 2019 included 55 first-episode schizophrenia (FES) cases and 53 healthy controls and genotyped them to assess how COMT contributes genetically to dorsolateral prefrontal cortex (DLPFC) changes in FES. The study found that the interactive effect between significant disease and COMT was mainly seen in the left DLPFC with the right precuneus, left anterior cingulated cortex, and right superior parietal gyrus. The resting-state functional connectivity (RSFC) of these DLPFC-related pathways was associated with affective blunting seen in FES patients with Val/Val genotype and not with carriers of the Met allele [18]. The study showed a pattern of gene action that was disease-dependent [18]. This finding may be helpful for the treatment of patients with Val/Val genotype to reduce the severity of symptoms.

There are multiple phenotypes of heroin use disorder, which might be associated with COMT genetic polymorphism. In a study done in 2021, 801 heroin users were included and divided into groups with four different phenotypes. The findings of the study suggested that subjects having the G (guanine) allele of rs769224 COMT variant were prone to consuming higher dosages of heroin every day and concluded that the findings could be helpful in the precise treatment of heroin use disorder [19]. A similar study of opioid use disorder conducted in 2019 investigated individual abuse potential depending on genetic variation. The study recruited 36 non-dependent adults with a history of exposure to medical prescription opioids and studied the subjective effects of oxycodone. The study found that "stimulated" effects of oxycodone were seen in COMT rs4680 variant, concluding that the euphoric and stimulating effects of oxycodone depend on genetic variation. Studies in the future should investigate the risk of developing opioid use disorder after medical prescription opioid exposure [20]. The sample size used in this study was very small. A study done in 2019 assessed the effect of COMT gene variants on the likelihood of opioid use and the dose of the opioid in 298 adults suffering from chronic pain and found that individuals with the Met/Met variant were more likely to use opioids than those with Val/Met variant. The dosage of opioids used was higher among the Val/Met and Met/Met genotypes than the Val/Val genotype [21]. This study could have future implications in opioid therapy for pain. Another related study done in 2019 recruited 42 adults with chronic pain receiving opioid therapy daily to determine the effect of COMT rs4680 SNP on hyperalgesia induced by opioids. Adults with Val/Met variant were found to be more hyperalgesic compared to Val/Val and Met/Met variants, using a measure of heat pain perception. The study concluded that the findings could help understand the influence of COMT on pain perception in daily opioid users [22]. These findings could further help in individualized patient-oriented treatment. 

In 2021, a study was done on 329 subjects from Lithuania that assessed the effect of Ankyrin Repeat and Kinase Domain Containing 1 - Dopamine Receptor D2 complex (ANKK1-DRD2) and COMT SNP on the risk of alcohol abuse. Having both genetic polymorphisms of COMT and ANKK1-DRD2 was associated with an elevated risk of hazardous use of alcohol [23]. Another study done in 2019 included 480 young adults to assess the likelihood of alcohol and drug use in people who experienced early-life adversity (ELA) in the presence of a COMT genotype. People with Val/Val genotype who experienced ELA had more significant cortisol responses compared to those with Met/Met or Val/Met genotype. In addition, ELA predicted earlier age of first alcoholic drink with a greater effect in Met allele than in Val/Val genotype. The study concluded that people with Val/Met or Met/Met variant of COMT who had exposure to ELA are more vulnerable to risky drinking behavior [24]. 

Methamphetamine increases dopamine levels in the prefrontal cortex, and its dependence affects executive function (EF) in individuals. In 2020, a study was done to assess how the different COMT genotypes alter the effects of methamphetamine dependence on EF. The study recruited 75 men with methamphetamine dependence (METH+) and 47 men with no dependence (METH-) and checked their cerebrospinal fluid (CSF) levels of dopamine and its metabolites. Lower dopamine levels were found in Met/Met METH+ men with a worse performance of EF. Among METH- men with Met/Met, the highest dopamine levels were seen, and these individuals performed EF better than Val/Met and Val/Val variants. It was concluded that slow clearance of dopamine worsens dopamine dysregulation associated with methamphetamine [25]. A similar study was done in 2019 with 149 subjects who performed three tests of EF. In people with methamphetamine dependence having the Val allele, EF is protected due to greater inactivation of dopamine, which suggests that vulnerability to executive dysfunction among methamphetamine users is driven by genetics. Additionally, COMT genotyping could lead to personalized management of neurocognitive effects among methamphetamine users [26].

Table 1 summarizes the above-mentioned findings. 

**Table 1:** Association of catechol-O-methyltransferase genotype polymorphism with psychiatric disorders COMT: catechol-O-methyltransferase; IL-10: Interleukin-10; DRD2: dopamine receptor D2; DLPFC: dorsolateral prefrontal cortex; G allele: guanine allele; Met: Methionine; Val: Valine; SNP:  single nucleotide polymorphism; ANKK1-DRD2: Ankyrin Repeat and Kinase Domain Containing 1 - Dopamine Receptor D2 complex; ELA: early life adversity; METH: methamphetamine

Author	Year of publication	Type of study	No. of subjects	Purpose of study	Results /conclusion
Wang J et al. [15]	2021	Observational	640	To assess the effect of interaction between COMT and IL-10 genotype polymorphisms on cognition in chronic schizophrenics.	COMT genotype polymorphism has an effect on language and schizophrenia. Interactive effects of genotypes of COMT and IL-10 were significant in schizophrenia patients.
Punchaichira TJ et al. [16]	2020	Observational	1677	To study the effect of rs1076560 (DRD2) and rs4680 (COMT) on tardive dyskinesia and cognition in schizophrenia patients.	COMT rs4680 variant was associated with tardive dyskinesia. Smokers with this variant had an increased risk of tardive dyskinesia. This variant had a positive association with emotional efficiency in schizophrenia patients.
Bosia M et al. [17]	2019	Observational	135	To study the effect of interaction between cannabis use and COMT genotype variants in schizophrenia patients.	Met carriers performed better on cognitive tasks – processing speed and verbal fluency.
Kang Y et al. [18]	2019	Observational	108	To assess how COMT contributes genetically to DLPFC changes in first-episode schizophrenia.	The interactive effect between significant disease and COMT was seen in the left DLPFC. Resting-state functional connectivity was associated with affective blunting in Val/Val genotype.
Deji C et al. [19]	2021	Observational	801	To study the association of COMT and Alpha-1-adrenergic receptor gene polymorphisms with multiple phenotypes of heroin use disorder.	The subjects having the G allele of rs769224 COMT variant were prone to consuming higher dosages of heroin every day.
Jones JD et al. [20]	2019	Observational	36	To assess the contribution of opioid- and dopamine-related genetic polymorphisms to the abuse liability of oxycodone.	The "stimulated" effects of oxycodone were seen in COMT rs4680 variant.
Hooten WM et al. [21]	2019	Observational	298	To assess the effect of COMT gene variants on the likelihood of opioid use and the dose of the opioid in adults suffering from chronic pain.	Individuals with the Met/Met variant were more likely to use opioids than those with Val/Met variant. The dosage of opioids used was higher among the Val/Met and Met/Met genotypes than the Val/Val genotype.
Hooten WM et al. [22]	2019	Observational	142	To assess the effect of COMT rs4680 SNP on hyperalgesia induced by opioids in adults with chronic pain receiving opioid therapy.	Adults with Val/Met variant were found to be more hyperalgesic compared to Val/Val and Met/Met variants.
Kaminskaite M et al. [23]	2021	Observational	329	To assess the effect of ANKK1-DRD2 and COMT SNP on the risk of alcohol abuse.	Having both genetic polymorphisms of COMT and ANKK1-DRD2 was associated with an elevated risk of hazardous use of alcohol.
Lovallo WR et al. [24]	2019	Observational	480	To assess the likelihood of alcohol and drug use in people who experienced ELA in the presence of a COMT genotype.	People with Val/Met or Met/Met variant of COMT who had exposure to ELA were more vulnerable to risky drinking behavior.
Saloner R et al. [25]	2020	Observational	122	To assess how the different COMT genotypes alter the effects of methamphetamine dependence on executive function.	Lower dopamine levels were found in Met/Met METH+ men with a worse performance of EF.
Cherner M et al. [26]	2019	Observational	149	To determine the effect of COMT genotype in methamphetamine-related executive dysfunction.	In people with methamphetamine dependence having the Val allele, executive function is protected.

Association of catechol-O-methyltransferase genotype polymorphism with neurological disorders

Dopamine plays a role in the pathophysiology of Parkinson's disease, and the COMT enzyme degrades dopamine. A study done in 2020 stated that the Val(158)Met variant of the COMT gene has recently been associated with a decline in cognition in Parkinson’s disease (PD). The study assessed the gray matter volume (GMV) in 120 PD patients with Val(158)Val variant of the COMT gene who had preserved cognition and found a widespread reduction in the GMV with atrophy in fronto-subcortical as well as the parieto-temporal regions of the cerebrum. A four-year follow-up showed cognitive decline associated with some of these regions and the study concluded that PD patients with a reduced GMV and a Val homozygous genotype are predisposed to a decline in cognition [12]. This finding warrants further investigation about the mechanism involved in the structural changes observed in the brain in PD patients, which could alter the therapeutic approach of PD. The progression of PD depends on changes in dopamine levels, which are affected by either the amount of damage to dopaminergic neurons or by the COMT enzyme activity, which is coded by the COMT genotype in individuals. In 2019, a study assessed the executive functions in 54 PD patients with different COMT gene variants and found that the PD patients with Val/Val genotype showed poorer performance in the set-shifting task as compared to the PD Met/Met and Val/Met genotypes. A cane-shaped curve was plotted to denote the dopamine level-response relationship for the set-shifting task. The study also found that COMT gene polymorphism influenced working memory in PD and it followed an inverted U curve relationship with dopamine levels which meant that poor working memory was seen in both higher and lower levels of dopamine. Lastly, the study found that the frontal dopamine level did not affect inhibition. The optimal level of dopamine to maximize executive functions might depend on the level of clinical progression of PD and the COMT gene variant present. Hence, this may direct the future of therapeutics of PD towards personalized medicine as different dosages might be needed depending on the different enzyme activity [27]. These studies included a very small number of PD patients, and new studies with larger sample sizes may be of benefit.

The Val(158)Met (rs4680) variant of the COMT gene has been associated with Alzheimer’s disease (AD). In a research study conducted in 2020, scientists assessed the association of this variant with the levels of cerebrospinal fluid (CSF) biomarkers of AD in 233 subjects. Amyloid-β42 (Aβ42) levels were significantly decreased in AD patients with GG in comparison to the AG COMT Val158Met variant. The levels of t-tau and p-tau181 were found to be increased in AD patients with AA (adenine-adenine) in comparison to the AG (adenine-guanine) COMT Val158Met genotype. The study concluded that the COMT Val158Met genotype could be investigated as a genetic marker of AD [28]. This study has important implications in the future of diagnostics in neuro medicine. In 2019, a study was done on Greek and Italian subjects, which included 156 AD patients and 301 healthy controls, to explore the effects of gene variants on psychiatric comorbidities in AD. The study found that the rs4680 variant of the COMT genotype was associated with AD, and the rs174696 variant of COMT was associated with depression in Greek AD patients. Thus, the study supported the role of the rs4680 variant in the disease pathogenesis of AD [29]. Since this research was conducted on a Greek and Italian subsample, this work should be replicated among other population samples as well. In 2019, a study investigated the association of single nucleotide polymorphisms in a few genes, including COMT, with the pathogenesis of AD and risk of dementia in 2857 postmenopausal women over the age of 65 years from the Women’s Health Initiative Memory Study (WHIMS). The results showed that the COMT gene was associated with probable dementia and that the studies involving candidate genes might help discover new pathways of structural and functional aging of the brain and understand variations in structure and function of the brain of different individuals. The study also suggested that the findings could help understand the role of candidate genes in cognition and cognitive impairment [30]. This, in turn, could lead to the discovery of effective interventions - both preventive and therapeutic. Table 2 summarizes the findings of the above-mentioned studies.

**Table 2:** Association of catechol-O-methyltransferase with neurological disorders COMT: catechol-O-methyltransferase; GMV: gray matter volume; PD: Parkinson's disease; Val: Valine; Met: Methionine; CSF: cerebrospinal fluid; AD: Alzheimer's disease; AA: adenine-adenine; AG: adenine-guanine; SNP: single nucleotide polymorphism

Author	Year of publication	Type of study	No. of subjects	Purpose of study	Results/conclusion
Sampedro F et al. [12]	2020	Observational	120	To determine the GMV in COMT Val/Val Parkinson’s disease (PD) patients with preserved cognition and its association with cognitive decline.	PD patients with a reduced GMV with a Val/Val genotype are predisposed to cognitive decline.
Fang YJ et al. [27]	2019	Observational	54	To assess the executive functions in PD patients with different COMT gene variants.	PD patients with Val/Val genotype showed poor performance in the set-shifting task. COMT gene polymorphism influences working memory in PD.
Babić Leko M et al. [28]	2020	Observational	233	To assess the association of COMT Val(158)Met genotype with the CSF biomarker level in Alzheimer’s disease (AD) patients.	The levels of t-tau and p-tau181 were found to be increased in AD patients with AA in comparison to the AG COMT Val158Met genotype.
Porcelli S et al. [29]	2019	Observational	457	To explore the effects of gene variants on psychiatric comorbidities in AD.	The rs4680 variant of the COMT genotype was associated with AD, and the rs174696 variant of COMT was associated with depression in Greek AD patients.
Driscoll I et al. [30]	2019	Observational	2857	To study the association of SNPs in a few genes, including COMT, with the pathogenesis of AD and risk of dementia in postmenopausal women.	COMT gene was associated with probable dementia.

Pharmacogenetic studies on the effect of COMT genotype polymorphism in neurological and psychiatric disorders

Aripiprazole is an atypical antipsychotic drug used in the treatment of schizophrenia. In a research study done in 2021, 98 patients with recent-onset schizophrenia were administered aripiprazole long-acting injectable (LAI) for three months, and genetic polymorphism of COMT along with two other genes was determined. Symptom severity was assessed, and it was found that usage of aripiprazole LAI improved cognitive functions. COMT Met/Met genotype and COMT interaction with methylenetetrahydrofolate reductase (MTHFR) gene were found to have a positive association with attention and executive functioning. The study concluded that COMT has the potential to be a biomarker of cognition in schizophrenic patients [31]. A longitudinal study done in 2020 looked at the association between two genotypes of COMT - rs4680 and rs4818 and response to treatment with antipsychotic drugs in 521 Caucasian schizophrenia patients. The study found a significant therapeutic response in schizophrenia patients with the COMT rs4680 A (adenine) allele and rs4680-rs4818 CA (cytosine-adenine) haplotype, who were treated with olanzapine, an atypical antipsychotic drug. The study concluded that the pharmacogenetic information gleaned through their data can help choose patients who will respond to olanzapine treatment in order to search for genetic markers of treatment outcome in patients suffering from schizophrenia [32]. 

A study conducted in 2020 investigated the association of gene polymorphisms with motor levodopa-induced complications (MLIC) in Parkinson’s Disease (PD). The study recruited 133 subjects and genotyped them for brain-derived neurotrophic factor (BDNF), dopamine transporter (DAT), and COMT genes. The study found a positive association between a combination of AG (adenine-guanine) BDNF, AG (adenine-guanine) DAT, and GG (guanine-guanine) COMT genotypes with MLIC and concluded that genetic biomarkers could help determine which PD patients are more prone to the long-term side effects of therapy with levodopa [33]. Another study in 2020 assessed the impact of variants in several genes on the development of levodopa-induced dyskinesia (LID) in 220 patients with idiopathic PD. COMT LL genotype was seen to increase the chances of developing LID, suggesting that polymorphism in the COMT genotype should be considered before the treatment of LID in PD patients [34]. Most of the studies of PD focus on the rs4680 variant of the COMT gene. A case-control study carried out in 2020 focused on 11 variants of the COMT gene to investigate their association with variability in response to levodopa therapy in 150 participants. SNPs other than rs4680 were found to have a possible association with variation in the daily dose of levodopa as well as susceptibility to the occurrence of dyskinesia after levodopa therapy. These findings could help optimize personalized dopaminergic drug therapies for PD patients [35]. A major limitation of these pharmacogenetic studies, which were based on levodopa usage for PD treatment in terms of COMT gene polymorphisms, was the small sample sizes. Therefore, future studies need to replicate them with larger sample sizes to increase the power of the conclusions. 

Patients have been found to show varied responses to the same antidepressive treatment. A meta-analysis was done in 2020 that included 11 studies involving 2845 subjects studied the association of COMT rs4680 variant with antidepressive treatment response and found that patients with the G allele of the COMT rs4680 gene responded better to electroconvulsive therapy (ECT). COMT rs4680 variant was significantly associated with antidepressive treatment, but this association varied amongst the individual studies. Patients with the GG or AG + GG variants responded better to ECT than patients with the AA (adenine-adenine) genotype. Only a small number of studies used ECT, and the authors saw this as a limitation of the study, which prompted them to suggest the need for further research to verify these findings [36]. A randomized control trial (RCT) done in 2020 investigated whether neuroplasticity-associated SNPs like COMT gene and other gene variants affect the efficacy of transcranial direct current stimulation (tDCS) or escitalopram therapy in major depression. The trial recruited 195 participants and divided them into three groups - escitalopram/tDCS-sham, tDCS/placebo-pill and placebo-pill/sham-tDCS. The results indicated non-significant dose-dependent effects of the Met allele and lower tDCS vs. placebo effects. The study noted the limitation of a small sample size resulting in non-significant findings [37]. In 2020, another RCT investigating the neurocognitive effects of tDCS treatment in 130 participants with unipolar or bipolar depression determined COMT and BDNF genotype polymorphisms as potential moderators of neurocognitive effects. Improvement in neurocognitive function was seen following tDCS treatment in patients with depression, and it was found that COMT rs4680 variant affected verbal fluency outcomes [38]. These findings could be helpful in personalized tDCS therapy as the gene variants have been shown to affect particular cognitive functions. 

Methadone is a drug used for maintenance treatment in patients dependent on opioids to reduce withdrawal symptoms. A study in 2020 investigated the association of COMT genotype variants with response to methadone maintenance treatment (MMT) in 820 Chinese opioid-dependent patients. Three polymorphisms of COMT were genotyped. The study found that patients with the TC (thymine-cytosine) or CC (cytosine-cytosine) genotypes of rs933271 variant had a higher chance of responding to MMT than patients with TT (thymine-thymine) allelic variant and the other two genotypes of COMT - rs4680 and rs737866 did not have an association with MMT response. These results could shed new light on methadone treatment in individuals and their sensitivity to methadone treatment, thus paving the way for a more effective therapeutic approach. However, the study lacked an assessment of multiple gene interactions and acknowledged the possibility of coexistence of more than one mental disorder [39]. Further research should be done taking these points into account and including larger sample sizes. Table 3 summarizes the findings of the above-mentioned studies.

**Table 3:** Pharmacogenetic studies showing the effect of catechol-O-methyltransferase genotype polymorphism in neurological and psychiatric disorders COMT: catechol-O-methyltransferase; Met: Methionine; MTHFR: methylenetetrahydrofolate reductase; A allele: adenine allele; CA: cytosine-adenine; MLIC: motor levodopa-induced complications; PD: Parkinson's disease; AG: adenine-guanine; GG: guanine-guanine; AA: adenine-adenine; LID: levodopa-induced dyskinesia; BDNF: brain-derived neurotrophic factor; DAT: dopamine transporter; Val: Valine; ECT: electroconvulsive therapy; tDCS: transcranial direct current stimulation; MMT: methadone maintenance treatment; TC: thymine-cytosine; CC: cytosine-cytosine

Author	Year of publication	Type of study	No. of subjects	Purpose of study	Results /conclusion
Peitl V et al. [31]	2021	Observational	98	To study how the long-acting aripiprazole influences cognitive functions in recent-onset schizophrenia.	COMT Met/Met genotype and COMT interaction with methylenetetrahydrofolate reductase (MTHFR) gene were found to have a positive association with attention and executive functioning.
Nikolac Perkovic M et al. [32]	2020	Observational	521	To study the association between catechol-O-methyltransferase (COMT) rs4680 and rs4818 haplotype with treatment response to olanzapine in patients with schizophrenia.	A significant therapeutic response to olanzapine was found in schizophrenia patients with the COMT rs4680 A allele and rs4680-rs4818 C-A haplotype.
Michałowska M et al. [33]	2020	Observational	136	To determine the association of gene polymorphisms with MLIC in Parkinson’s Disease (PD).	A positive association was found between a combination of AG BDNF, AG DAT, and GG COMT genotypes with MLIC.
Dos Santos EUD et al. [34]	2020	Observational	220	To study the impact of variants in several genes on the development of levodopa-induced dyskinesia (LID) in patients with idiopathic PD.	COMT LL genotype was seen to increase the chances of developing LID.
Zhao C et al. [35]	2020	Observational	150	To investigate the association of COMT genotype variants with variability in response to levodopa therapy in PD patients.	Single nucleotide polymorphisms other than rs4680 were found to have a possible association with variation in the daily dose of levodopa as well as susceptibility to the occurrence of dyskinesia after levodopa therapy.
Tang Z et al. [36]	2020	Meta-analysis	2845	To study the association between COMT gene Val108/158Met and antidepressive treatment response.	COMT rs4680 variant was found to be significantly associated with antidepressive treatment. Patients with the GG or AG + GG variants responded better to ECT than patients with AA genotype.
Brunoni AR et al. [37]	2020	Randomized control trial	195	To investigate whether neuroplasticity-associated SNPs like COMT gene and other gene variants affect the efficacy of tDCS or escitalopram therapy in major depression.	The results indicated non-significant dose-dependent effects of the Met allele and lower tDCS vs. placebo effects.
McClintock SM et al. [38]	2020	Randomized control trial	130	To assess the neurocognitive effects of transcranial direct current stimulation (tDCS) in unipolar and bipolar depression.	COMT and BDNF genotype polymorphisms were determined as potential moderators of neurocognitive effects. COMT rs4680 variant was found to affect verbal fluency outcomes.
Duan L et al. [39]	2020	Observational	820	To study the association of COMT gene polymorphisms with response to MMT among opioid-dependent patients.	Patients with the TC or CC genotypes of the rs933271 variant had a higher chance of responding to MMT.

## Conclusions

This review focused on the association of the various COMT genotype polymorphisms in neurological and psychiatric disorders, emphasizing its therapeutic implications, especially towards personalized medicine. We have explored how the susceptibility to neurological and psychiatric diseases, their specific symptoms, and therapeutic interventions is dependent on genetics. In schizophrenia patients, significant interactive effects of genotypes of COMT and IL-10 were seen, and the COMT rs4680 variant was associated with tardive dyskinesia. COMT gene polymorphism influenced working memory in Parkinson’s disease. Individuals with the Met/Met variant were more likely to use opioids than those with the Val/Met variant of the COMT gene. Having both genetic polymorphisms of COMT and ANKK1-DRD2 was associated with an elevated risk of hazardous use of alcohol. We also reviewed several pharmacogenetic studies. A significant therapeutic response to olanzapine was found in schizophrenia patients with the COMT rs4680 A allele and rs4680-rs4818 CA haplotype. COMT rs4680 variant was found to be significantly associated with antidepressive treatment. Patients with the TC or CC genotypes of rs933271 variant had a higher chance of responding to methadone maintenance treatment among opioid-dependent patients. These findings prove that there is a huge variation in individual response to disease pathophysiology and therapeutic strategies based on genetic polymorphisms, which paves the way for personalized medicine. It is a well-known fact that the prognosis of various neurological and psychiatric disorders is poor. Focusing on targeted therapeutic strategies can improve the quality of life of patients and decrease the cost of care of patients. Most of the studies we included in this review were newer observational studies with small sample sizes, and we also excluded studies with subjects below the age of 18. We recommend future researchers conduct more randomized control trials (RCTs) to investigate the therapeutic role of COMT genotype polymorphism in neurological and psychiatric disorders.
